# Novel Function of Rev-erbα in Promoting Brown Adipogenesis

**DOI:** 10.1038/srep11239

**Published:** 2015-06-10

**Authors:** Deokhwa Nam, Somik Chatterjee, Hongshan Yin, Ruya Liu, Jeongkyung Lee, Vijay K. Yechoor, Ke Ma

**Affiliations:** 1 Center for Diabetes Research, Department of Medicine, Houston Methodist Research Institute, Houston, TX, 77030; 2 Department of Cardiovascular Medicine, Second Affiliated Hospital, Hebei Medical University, Shijiazhuang, Hebei, 050017, China; 3 Diabetes and Endocrinology Research Center, Department of Medicine, Baylor College of Medicine, Houston, TX, 77030.

## Abstract

Brown adipose tissue is a major thermogenic organ that plays a key role in maintenance of body temperature and whole-body energy homeostasis. Rev-erbα, a ligand-dependent nuclear receptor and transcription repressor of the molecular clock, has been implicated in the regulation of adipogenesis. However, whether Rev-erbα participates in brown fat formation is not known. Here we show that Rev-erbα is a key regulator of brown adipose tissue development by promoting brown adipogenesis. Genetic ablation of *Rev-erbα* in mice severely impairs embryonic and neonatal brown fat formation accompanied by loss of brown identity. This defect is due to a cell-autonomous function of Rev-erbα in brown adipocyte lineage commitment and terminal differentiation, as demonstrated by genetic loss- and gain-of-function studies in mesenchymal precursors and brown preadipocytes. Moreover, pharmacological activation of Rev-erbα activity promotes, whereas its inhibition suppresses brown adipocyte differentiation. Mechanistic investigations reveal that Rev-erbα represses key components of the TGF-β cascade, an inhibitory pathway of brown fat development. Collectively, our findings delineate a novel role of Rev-erbα in driving brown adipocyte development, and provide experimental evidence that pharmacological interventions of Rev-erbα may offer new avenues for the treatment of obesity and related metabolic disorders.

The nuclear receptor, Rev-erbα (Nr1d1), is a ligand-regulated transcription repressor that possesses diverse physiological functions ranging from metabolic regulation, cellular differentiation to circadian rhythm^1^,^2^. As a constitutive repressor of transcription, Rev-erbα binds to DNA response element shared with the retinoid acid receptor-related orphan receptor alpha (RORα), known as RevRE/RORE, and recruit corepressors such as nuclear receptor corepressor (NCoR) to inhibit target gene transcription^3^,^4^,^5^.

Recently, Rev-erbα has been shown to be a key component of a regulatory loop within the molecular clock circuit^6^,^7^. *Rev-erbα* represses the core clock activator, Brain and Muscle Arnt-like 1 (Bmal1), and together with RORα, it generates the rhythmic expression of *Bmal1* that drives the circadian clock cycle^6^. As *Rev-erbα* itself is under the direct transcriptional activation of *Bmal1*, these mechanisms form a reinforcing loop of the circadian clock machinery^7^. Accumulating evidence demonstrate that the molecular clock is intimately linked with metabolic processes, and disruption of this temporal regulation leads to metabolic disorders such as obesity and insulin resistance^8^,^9^,^10^. However, the precise mechanisms mediating clock effect on metabolism have not been fully understood. Given the involvement of *Rev-erbα* in diverse metabolic processes, it is possible that certain metabolic effects of circadian misalignment may involve altered *Rev-erbα* function.

The brown adipose tissue (BAT) is a metabolically active organ distinguished by its unique capacity for adaptive thermogenesis in response to cold or adrenergic stimuli^11^. It has been increasingly recognized that, in addition to its critical role in maintenance of body temperature under physiological conditions or cold-stress^11^, the energy-dissipating capacity of BAT is an important regulatory component of whole-body energy balance^12^,^13^. An inverse correlation of the amount of functional BAT with body-mass index was found in obese subjects^13^,^14^,^15^, suggesting that its energy-dissipating function may impact the development of obesity. Similar to white adipocyte differentiation^16^, formation of mature brown adipocytes, brown adipogenesis, requires the adipogenic cascade of sequential activation of adipogenic factors, CEBPβ, PPARγ and CEBPα. This shared adipogenic pathway dictates the phenotypic overlap between BAT and white adipose tissue (WAT). However, compare to the energy-storage function of WAT, BAT disperse chemical energy derived from oxidative phosphorylation as heat to maintain body temperature instead of ATP generation^11^. The thermogenic function of BAT is mediated by the proton channel in the inner mitochondrial membrane, uncoupling protein-1 (UCP-1)^11^. Due to this distinct functional requirement, mature brown adipocyte formation entails the coordination of a brown-specific thermogenic gene program and mitochondrial biogenesis^17^. Recent lineage tracing studies indicate that although BAT shares a mesodermal origin with WAT, it diverges from a *Myf5*^*+*^ myogenic lineage under the instructive signal of *Prdm16*^18^, an early determination factor of brown adipocyte precursors.

*Rev-erbα* has been shown to regulate adipogenesis of white adipocyte^19^,^20^,^21^. *Rev-erbα* mRNA expression is induced during adipogenic differentiation^19^ and overexpression of *Rev-erbα* enhances adipogenesis as a downstream target of PPARγ^20^. Interestingly, recent report demonstrates that *Rev-erbα* polymorphisms are associated with obesity in humans^22^. However, whether *Rev-erbα* functions in the brown adipogenic pathway or brown fat development has not been explored. Based on the shared adipogenic cascade requisite for BAT and WAT formation, we hypothesize that Rev-erbα may play a role in brown adipocyte development. In the current study, we employed genetic animal models, loss- and gain-of-function cellular studies, and pharmacological tools to investigate the physiological functions of *Rev-erbα* in brown adipocyte differentiation and BAT development.

## Results

### Loss of Rev-erbα impairs BAT development

**Figure 1:**
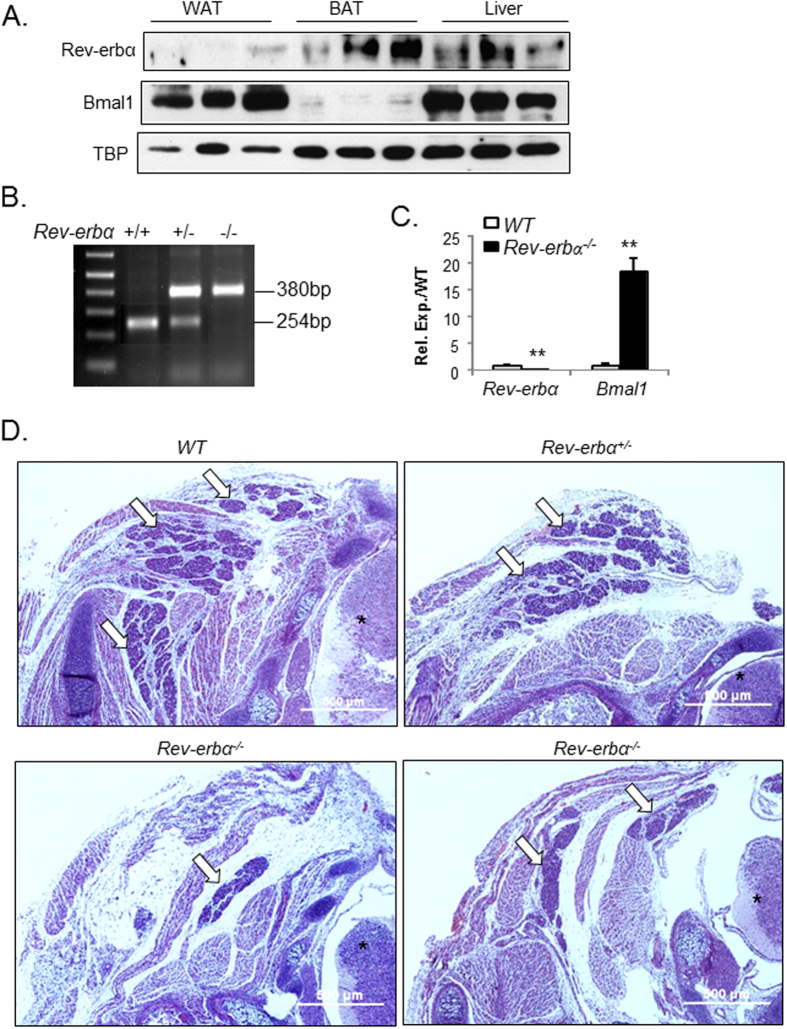
Genetic ablation of *Rev-erbα* impairs brown adipose tissue development. (**A**) Cropped images of immunoblot analysis of Rev-erbα and Bmal1 protein in normal control mice brown fat under ambient temperature at 5 pm. (**B**) PCR genotyping of wild-type, *Rev-erba*^*+/−*^ and *Rev-erbα*^*−/−*^ mice as indicated by the 380 bp null and 254 bp wild-type alleles. (**C**) RT-qPCR analysis showing absence of *Rev-erbα* expression and up-regulation of *Bmal1* in Rev*-erbα*^*−/−*^ mice brown fat (n = 5-6). **: P < 0.01 *Rev-erbα*^*−/−*^ vs. WT. (**D**) Representative images of H/E histology of interscapular brown fat (one side, 4X) of E18.5 embryos. Serial sections through the neck were obtained, and images of the same anatomical position were compared in *Rev-erbα*^*−/−*^, *Rev-erbα*^*+/−*^ and WT mice. Arrows denote brown fat and asterisks indicate spinal cord.

To study the function of *Rev-erbα* in brown fat, we first examined its abundance in brown and white adipose tissue. Rev-erbα protein is highly enriched in BAT as compared to its low expression in WAT, at a level that is comparable to the liver (Fig. 1A). In contrast, the *Rev-erbα* target gene in the molecular clock circuit, Bmal1, display an opposite distribution pattern in adipose tissues with higher level in WAT than BAT. To investigate the *in vivo* function of *Rev-erbα* in brown fat development, we generated *Rev-erbα*-null (*Rev-erbα*^*−/−*^) mice using gene-targeted ES cell clone obtained from the KOMP consortium, with the null allele identified as a 380 bp PCR amplification (Fig. 1B). The homozygote null mice are viable, but adult mice show reduced fertility, as reported previously^23^. The brown fat of *Rev-erbα*^*−/−*^ mice display complete ablation of *Rev-erbα* (Fig. 1C), while mRNA level of its direct target gene, *Bmal1*, is markedly induced as compared to wild-type controls (WT), as expected. Next, we examined the effects of loss of *Rev-erbα* function on BAT development in embryonic and neonatal stages in WT, heterozygote and homozygote mutants. As BAT forms late during development with fully mature features detected after E16.5^24^, we first analyzed BAT formation at E18.5 embryos by haematoxylin and eosin staining. BAT in WT mice at this stage of development display fully organized, densely stained structures with three separate lobes in between the dermis and underlying muscle layer, as shown in Fig. 1D. In contrast, the size of interscapular BAT in *Rev-erbα*^*−/−*^ mice is markedly reduced as compared to that of the WT littermates (Fig. 1D), with large part of the structure replaced by white adipose tissue. In addition, the structural organization of these brown fat pads with the underlying muscular layer is severely disrupted, with skeletal muscle interspersed within the brown fat. BAT of *Rev-erbα*^*+/−*^ embryos are also smaller, but their juxtaposition with adjacent muscle is largely maintained. Therefore, the loss of *Rev-erbα in vivo* markedly impairs the formation, structural integrity and characteristics of BAT.

**Figure 2:**
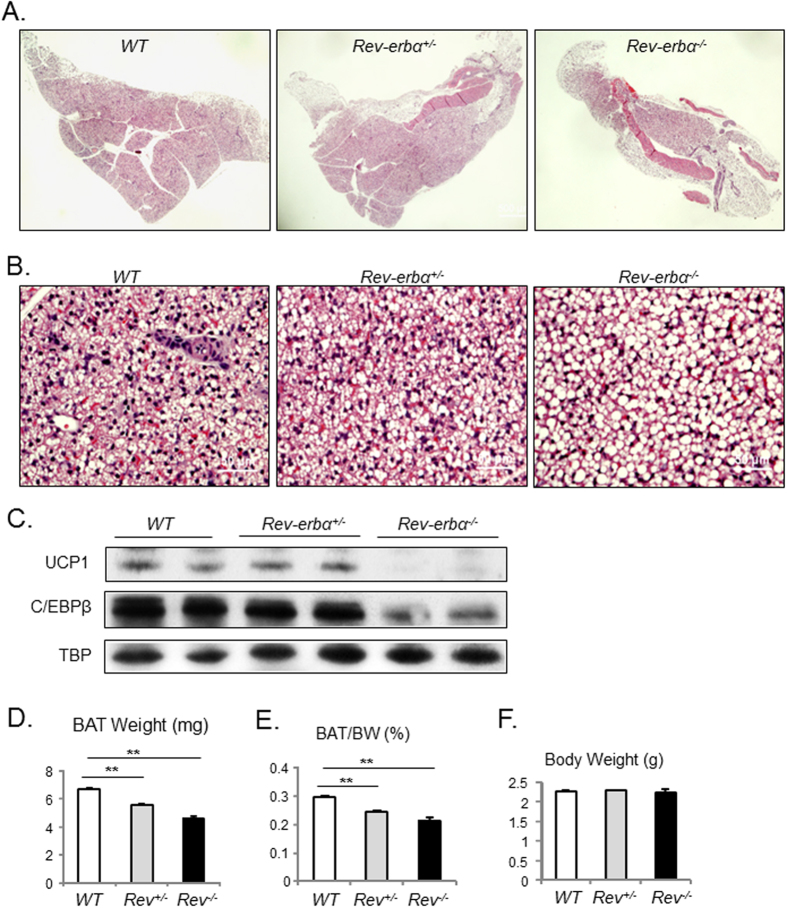
Loss of *Rev-erbα* reduces brown adipose tissue formation in neonatal mice. (**A**,**B**) Representative images of H/E histology analysis of postnatal day 5 (P5) *Rev-erbα*^*−/−*^, *Rev-erbα*^*+/−*^ and WT mice at 4X (**A**), and 20X (**B**) magnification. (**C**) Cropped images of immunoblot of brown adipogenic markers, UCP-1 and C/EBPβ, in BAT (n = 6/group, pooled sample of 3 mice/lane). Full-length images are shown in Fig. S6. (**D–F**) BAT tissue weight (**D**), ratio to total body weight (**E**), and total body weight (**F**) of P5 neonates (n = 6–8/group). **: P < 0.01 *Rev-erbα*^*−/−*^ or *Rev-erbα*^*+/−*^ *vs.* WT.

We further examined BAT formation in early postnatal development of day 5 (P5), which allows us access to tissues and quantitative analysis. Similar substantially reduced size and disrupted structure of BAT is evident in the *Rev-erbα*^*−/−*^ mice through examination of one side of the interscapular fat pad (Fig. 2A), with *Rev-erbα*^*+/−*^ affected to a lesser extent. Reduction of the *Rev-erbα*^*−/−*^ BAT size is accompanied by diminished mitochondrial-rich cytoplasmic staining characteristic of brown fat and its replacement by increased lipid droplets as compared to controls (Fig. 2B). Analysis of total protein in BAT reveals nearly absence of UCP-1 expression and marked reduction of C/EBPβ in these mice, corroborating the histological observation of loss of brown fat identity (Fig. 2C). Likely due to impaired brown fat development, the BAT mass as determined by its tissue weight (Fig. 2D), or tissue to body weight ratio (Fig. 2E) is ~16% and ~31% lower in neonatal *Rev-erbα*^*+/−*^ and *Rev-erbα*^*−/−*^ mice than that of the littermate controls, respectively. Their body weight at this developmental stage is not altered (Fig. 2F). Similar to what we found in embryonic development and neonatal mice, brown fat weight in 10 weeks old adult *Rev-erbα*^*+/−*^ and *Rev-erbα*^*−/−*^ mice remains to be significantly lower (Fig. S1A).

Interestingly, in addition to impaired BAT formation, adult *Rev-erbα*^*−/−*^ display ~40% reduction of WAT mass relative to the WT, as indicated by measured epididymal fat pad weight (Fig. S1B) or NMR analysis of total body fat (Fig. S1C & S1D). In addition, NMR analysis reveals a significant reduction of total lean mass in Rev^*−/−*^ mice, but not in Rev^*+/−*^ mice (Fig. S1E). The body weight of adult *Rev-erbα*^*−/−*^ mice are also significantly lower than that of the WT controls (Fig. S1F), whereas their heart weights are not affected (Fig. S1G). As lean mass accounts for over 80% of total body weight, the reduced lean weight likely contributed to the lower body weight observed in Rev^*−/−*^ mice together with lower fat mass. The reduced amount of white adipose tissue observed in adult Rev^*−/−*^ mice is consistent with previously reported Rev-erba positive regulation of adipogenesis^20^,^25^, and shRNA silencing of *Rev-erbα* in 3T3-L1 cells further demonstrates this function (Fig. S2). Interestingly, despite the reduction of brown adipose tissue in adult *Rev-erbα*^*−/−*^ mice, the H/E histology is largely comparable to that of the WT (Fig. S3A), and cold tolerance test reveals similar responses (Fig. S3B). Furthermore, gene expression analysis indicate induction of UCP-1 mRNA in *Rev-erbα*^*−/−*^ mice, although brown adipogenic markers (*Myf5, Pparg, Fabp4*), and mitochondrial genes (*Pgc1a and Cox7a1*) are down-regulated (Fig. S3C). These intriguing findings suggest that despite the marked impairment of brown adipose tissue development during embryonic and neonatal stages, adult *Rev-erbα*^*−/−*^ mice do generate functional brown fat.

### Silencing of Rev-erbα inhibits mesenchymal precursor brown lineage commitment and differentiation

**Figure 3:**
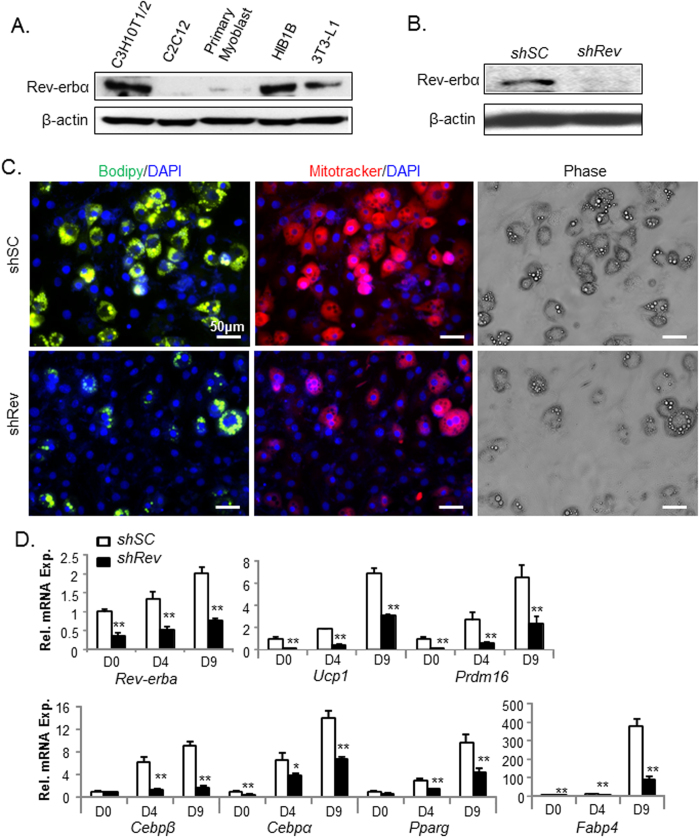
Effect of stable *Rev-erbα* knockdown on C3H10T1/2 brown adipogenic differentiation. (**A**) Cropped image of Rev-erbα immunoblot in mesodermal lineage cell lines. Full-length images are shown in Fig. S7. (**B**) Near absence of Rev-erbα protein in C3H10T1/2 cells with stable silencing of Rev-erbα (*shRev*) as compared to scrambled control (*shSC*). (**C**) Representative images of C3H10T1/2 lipid accumulation as shown by BODIPY staining, mitochondrial staining by Mitotracker, and phase contrast at day 9 of differentiation under a brown adipocyte induction condition. (**D**) RT-qPCR analysis of brown adipocyte marker gene expression in *shSC* and *shRev* 10T1/2 cells during brown differentiation induction time course at 0, 4 and 9 days of differentiation. *, **: P < 0.05 and 0.01 *shRev* vs. *shSC*.

Based on the finding that *Rev-erbα* promotes BAT development, we next explored whether it directly participates in brown adipogenesis in a cell-autonomous manner using genetic loss- and gain-of-function studies in brown adipocyte progenitors. Brown adipocytes arise from mesenchymal precursors that first commit to a brown progenitor fate and subsequently undergo terminal differentiation^26^. Initial analysis of Rev-erbα protein level in mesenchymal precursors and brown preadipocytes, as compared to white preadipocytes and myoblasts, indicate that it is more abundant in C3H10T1/2 (10T1/2) mesenchymal stem cells and HIB1B brown preadipocytes (Fig. 3A), two cell types that are capable of differentiating into mature brown adipocytes under specific induction conditions. Using stable transfection of a retroviral short hairpin RNA (shRNA) construct targeting *Rev-erbα* (*shRev*), we generated stable knockdown clones of shRev-expressing 10T1/2 mesenchymal precursors, which results in near absence of Rev-erbα protein as compared to cells expressing scrambled control shRNA (*shSC*) (Fig. 3B). When subjected to a brown adipocyte induction cocktail, scrambled control cells display robust progression to mature adipocyte, with ~48% exhibiting typical round morphology and muti-locular lipid accumulation as indicated by phase-contrast images (Fig. 3C). In comparison, *Rev-erbα* silencing markedly reduces the percentage of cells with mature adipocyte morphology to ~22%. BODIPY staining of neutral lipids accumulation as an index of mature differentiation confirms the reduction of lipid droplets in *shRev* cells. Moreover, MitoTracker Red staining of functional mitochondria in live cells is substantially weaker in *Rev-erbα*-deficient cells than that of the *shSC*. The observed lower mitochondrial abundance suggests that loss of *Rev-erbα* impairs mitochondrial biogenesis associated with mature brown adipocyte differentiation. Furthermore, gene expression analysis at beginning, day 4 and 9 of differentiation demonstrates a markedly suppressed brown adipogenic gene program (Fig. 3D). The induction of brown-specific differentiation markers, *Ucp-1* and *Prdm16*, are significantly attenuated in *shRev* cells as compared to their robust expression (~7-8 fold induction) in the controls. The lack of induction of adipogenic genes, *Cebpβ, Cebpα, Pparγ* and *FABP4* during the differentiation time course indicates suppressed adipogenesis (Fig. 3D). We also found a moderate ~2-fold up-regulation of *Rev-erbα* mRNA level at day 9 of brown adipogenic induction, with significantly attenuated expression in the *shRev* cells. Collectively, these effects of *Rev-erbα* deficiency in 10T1/2 cells indicate that it modulates the lineage commitment and differentiation of mesenchymal precursor cells into brown adipocytes.

### Rev-erbα promotes brown preadipocyte terminal differentiation

**Figure 4:**
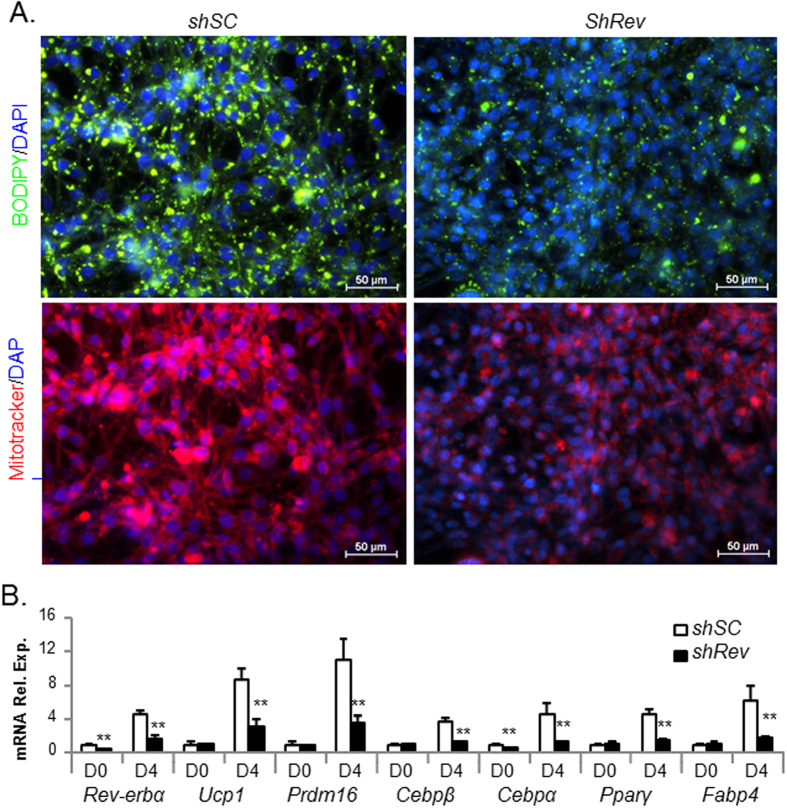
Effect of stable *Rev-erbα* knockdown on HIB1B terminal differentiation. (**A**) Representative images of lipid accumulation as shown by BODIPY staining and mitochondrial staining by Mitotracker in day 6-differentiated *shRev* and *shSC* HIB1B cells. (**B**) RT-qPCR analysis of brown adipocyte marker gene expression in day 4-differentiated *shSC* and *shRev* cells. *, **: P < 0.05 and 0.01 *shRev* vs. *shSC*.

Next, we determined the role of Rev-erbα in terminal differentiation stage of brown adipogenesis through gain- and loss-of-function studies in HIB1B brown preadipocytes. Similar to the phenotype observed with stable *Rev-erbα* shRNA depletion in mesenchymal precursors, inhibition of Rev-erbα in committed brown preadipocytes markedly suppresses lipid accumulation and mitochondrial abundance at day 6 of differentiation, as indicated by reduced BODIPY and MitoTracker staining relative to scrambled control cells (Fig. 4A). This significantly attenuated differentiation is accompanied by marked suppression of brown specific marker gene expression (*Ucp-1*, *Dio2* and *Prdm16)*, and adipogenic factors (*Cebpβ*, *Cebpα* and *Pparγ)* (Fig. 4B). Stable Rev-erbα depletion in these cells achieved ~70% reduction of *Rev-erbα* transcript.

**Figure 5:**
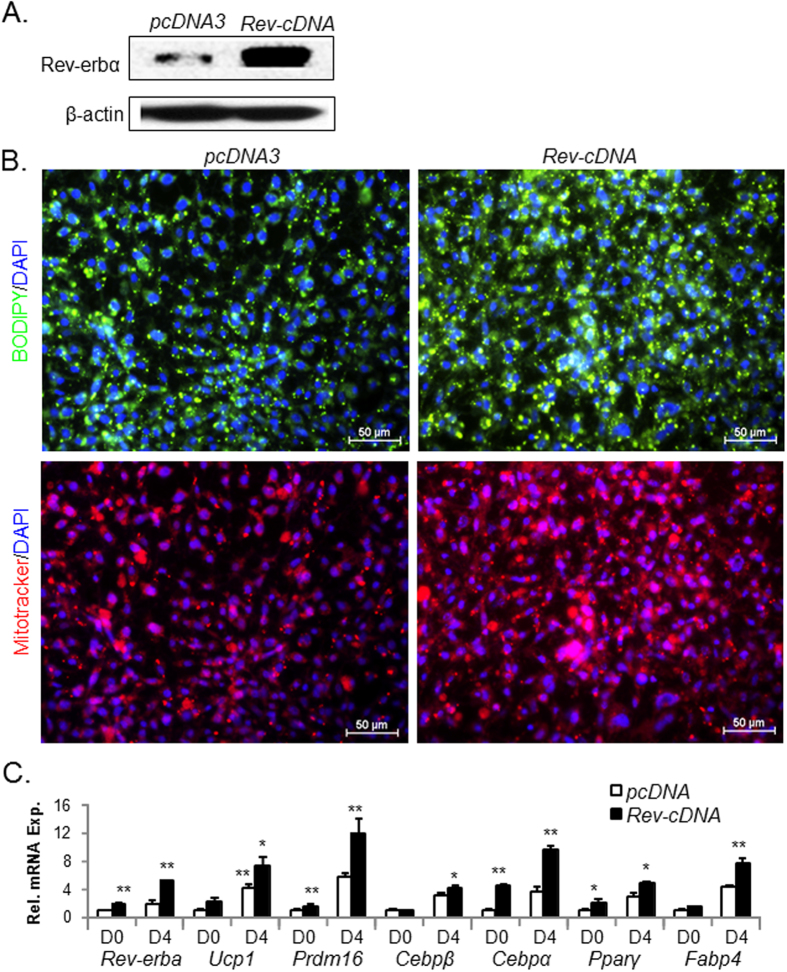
Forced expression of *Rev-erbα* promotes HIB1B terminal differentiation. (**A**) Cropped image of *Rev-erbα* immunoblot analysis of HIB1B cells with stably expression of vector control (pcDNA3) or *Rev-erbα* cDNA (Rev-cDNA) construct. (**B**) Representative images of lipid accumulation as shown by BODIPY staining and mitochondrial staining by MitoTracker in day 6-differentiated pcDNA3 control or Rev-cDNA-expressing cells. (**C**) RT-qPCR analysis of brown adipocyte marker gene expression in day 4-differentiated pcDNA3 or Rev-cDNA cells. *, **: P < 0.05 and 0.01 or Rev-cDNA vs. pcDNA3.

Using stable expression of a *Rev-erbα* cDNA plasmid (Rev-cDNA) in HIB1B cells, we tested the effect of *Rev-erbα* overexpression on brown adipocyte terminal differentiation. Forced expression of *Rev*-cDNA markedly elevated *Rev-erbα* protein level over vector control, pcDNA3 (Fig. 5A). Consistent with its protein expression, *Rev-erbα* transcript is increased ~6-fold in *Rev*-cDNA-expressing cells as indicated by RT-qPCR analysis (Fig. 5C). BODIPY and MitoTracker staining to assess mature differentiation demonstrate increased lipid accumulation and mitochondrial abundance in *Rev*-cDNA-expressing cells at day 6 of differentiation than controls, indicating that ectopic expression of *Rev-erbα* enhances terminal differentiation (Fig. 5B). Of note, at the same differentiation stage, the pcDNA3 vector control display less lipid accumulation and mitochondrial staining than scrambled shRNA control shown in Fig. 5B, suggesting a slight inhibitory effect of pcDNA3 vector on brown differentiation. Gene expression analysis of *Rev-erbα*-overexpressing HIB1B cells further demonstrates marked induction of brown adipocyte-specific markers over vector controls, together with moderate up-regulation of genes involved in adipogenesis (Fig. 5C).

**Figure 6:**
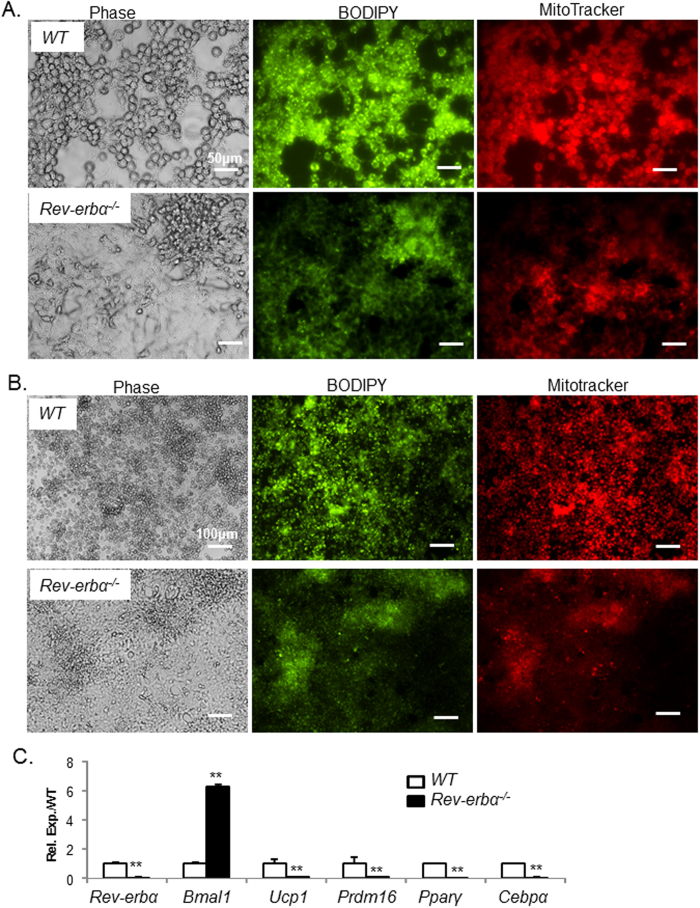
*Rev-erbα-null* primary brown preadipocytes display impaired differentiation. (**A**,**B**) Representative images of phase contrast morphology, lipid staining by BODIPY and mitochondrial staining by MitoTracker of immortalized wild-type and *Rev-erbα*^*−/−*^ primary brown preadipocytes at 10X (**A**), and 20X (**B**) magnification after 2 days of differentiation. (**C**) RT-qPCR analysis of brown adipogenic marker gene expression in wild-type and *Rev-erbα*^*−/−*^ brown preadipocytes (n = 3). **: P < 0.01 *Rev-erbα*^*−/−*^ *vs.* WT.

To assess the impact of complete loss of *Rev-erbα* function on brown preadipocyte differentiation, we isolated primary brown preadipocytes from WT and *Rev-erbα*^*−/−*^ mice and generated immortalized cell clones using SV40 large T antigen retroviral transduction. As shown in Fig. 6A,B immortalized WT primary brown preadipocytes exhibit rounded mature brown adipocyte morphology with robust lipid accumulation and mitochondrial formation upon brown adipogenic induction. In sharp contrast, differentiation of *Rev-erbα*^*−/−*^ brown preadipocytes is severely blocked, as they fail to progress to mature adipocytes and display barely detectable amount of lipid and mitochondria staining. Nearly abolished brown adipogenic marker gene induction in *Rev-erbα*^*−/−*^ cells relative to WT controls further confirms the severe defect in differentiation (Fig. 6C). Taken together, these findings indicate that Rev-erbα positively regulates terminal differentiation in lineage-committed brown preadipocytes.

### Pharmacological interventions of Rev-erbα activity modulates brown adipogenesis

**Figure 7:**
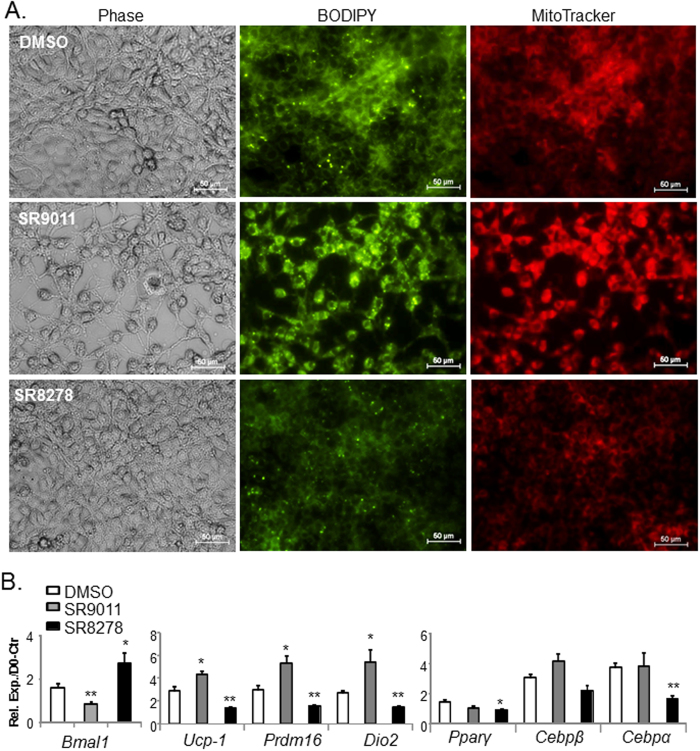
Rev-erbα agonist, SR9011, promotes, whereas antagonist, SR8278, inhibits brown adipocyte differentiation. (**A**) Representative images of phase contrast morphology, lipid (BODIPY) and mitochondrial (Mitotracker) staining of day 2–differentiated HIB-1B cells in the presence of DMSO (0.1%), SR9011 (10 uM in DMSO) or SR8278 (10 uM in DMSO) treatment. Indicated Rev-erbα ligand treatments were started 8 hours prior to induction of differentiation and maintained throughout differentiation. (**B**) RT-qPCR analysis of brown adipocyte differentiation marker genes in the presence of indicated Rev-erbα ligands after 2 days of differentiation (n = 3). *, **: P < 0.05 or 0.01 ligand treated *vs.* DMSO.

As a ligand-dependent nuclear receptor, the transcriptional activity of Rev-erbα is amenable to regulation by small molecule ligands, and both its agonist, SR9011, and antagonist, SR8278, display metabolic effects^27^,^28^,^29^. Therefore, we tested the effect of pharmacological activation and blockade of Rev-erbα activity on brown adipocyte differentiation using SR9011 and SR8278, respectively. In line with results from genetic interventions, *Rev-erbα* activation by SR9011 enhances, whereas its inhibition by SR8278 impairs brown preadipocyte terminal differentiation in comparison to DMSO vehicle control, as indicated by lipid and mitochondrial staining shown in Fig. 7A,B. Repression and induction of *Bmal1* mRNA in response to SR9011 and SR8278, respectively, confirms the modulation of *Rev-erbα* activity by these ligands in brown adipocytes (Fig. 7B). SR8278 suppression of brown adipocyte marker genes expression further validates the findings on differentiation as assessed by morphological progression (Fig. 7B). Interestingly, although SR9011 significantly up-regulates brown-specific markers, its effect on adipogenic factors are not evident. Together, these results demonstrate that modulation of Rev-erbα activity by pharmacological means is sufficient to impact brown adipogenesis.

### Rev-erbα promotes brown adipogenesis through negative regulation of the TGF-β cascade

**Figure 8:**
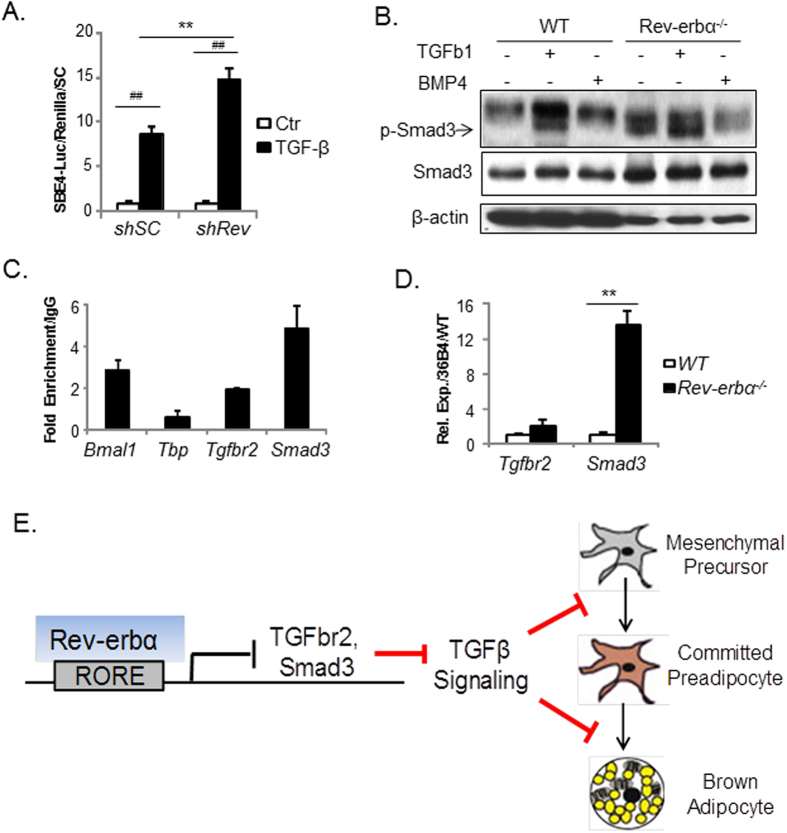
*Rev-erbα* suppresses TGF-β signaling via transcriptional control of key components of this pathway. (**A**) Analysis of TGF-β activity in *shSC* and *shRev* HIB-1B cells as assessed by TGF-β-responsive SBE4-Luc luciferase reporter assay (n = 4). ##: P < 0.01 TGF-β-treated vs. control; **: P < 0.01 *shRev* vs. *shSC*. (**B**) TGF-β signaling as analyzed by Smad3 phosphorylation in response to ligand treatment in WT and *Rev-erbα*^*−/−*^ brown preadipocytes, with cropped images of immunoblot analysis shown and full-length images included in Fig. S8. shown. Smad3 phosphorylation is assessed 1 hour after indicated ligand treatment (TGF-β, 10 ng/ml, BMP4 100 ng/ml). (**C**) ChIP-qPCR analysis of *Rev-erbα* occupancy of identified RORE sites in TGF-β pathway gene promoters. *Rev-erbα* binding in *Bmal1* intron was used as a positive control, and non-specific sites on *Tbp* promoter was used as negative control. Values were calculated as fold enrichment of percentage of total input normalized to IgG (n = 4). (**D**) RT-qPCR analysis of TGF-β pathway gene expression in immortalized WT and *Rev-erbα*^*−/−*^ brown preadipocytes (n = 3). (**E**) Proposed model of Rev-erbα control of brown adipocyte lineage commitment and terminal differentiation through transcriptional regulation of the TGF-β pathway.

TGF-β cascade is a key developmental signal that suppresses brown adipogenesis^30^,^31^,^32^. Interestingly, molecular clock components in epidermal stem cells regulate various signaling cascades, including the TGF-β pathway^33^,^34^, and a recently published ChIP-Seq dataset revealed *Rev-erbα* binding peaks on certain TGF-β pathway gene regulatory regions^28^. We thus tested whether *Rev-erbα* could modulate TGF-β signaling to impact brown adipogenesis. Using the TGF-β-responsive luciferase reporter, SBE4-Luc, to assess TGF-β pathway activity, we found significantly increased reporter activity in *Rev-erbα*-deficient 10T1/2 cells as compared to SC control upon TGF-β stimulation, indicating that loss of *Rev-erbα* function promotes TGF-β signaling transduction (Fig. 8A). Smad3 phosphorylation in response to TGF-β activation ultimately transduces TGF-β signaling to the nucleus to modulate gene transcription^35^. Therefore, we examined Smad3 phosphorylation in WT and *Rev-erbα-null* brown preadipocytes. Robust Smad3 phosphorylation is detected in WT cells only in response to TGF-β1, as expected. In contrast, *Rev-erbα-null* cells already display a moderate level of Smad3 phosphorylation at basal condition that is enhanced by TGF-β1 treatment (Fig. 8B). Non-responsiveness of Smad3 to BMP4 stimulation validates the specificity of TGF-β1 signaling. Interestingly, total Smad3 protein level is increased in *Rev-erbα-null* preadipocytes, indicating that *Rev-erbα* impacts Smad3 expression. To address potential direct transcriptional regulation of *Rev-erbα* on genes of the TGF-β signaling pathway, we screened for RORE elements, in −2 kb and first intron of their regulatory regions to identify putative *Rev-erbα* binding sites. Among the genes screened, including the ligands *Tgfb1* and *Tgfb2*, the receptors *Tgfbr1* and *Tgfbr2*, and signal transducer *Smad3*, *Rev-erbα* response elements were identified in promoter regions of *Tgfbr2* and *Smad3*, as listed in Fig S3. Using specific primers flanking these identified sites (Fig. S4), chromatin immunoprecipitation qPCR (ChIP-qPCR) analysis detects *Rev-erbα* enrichment on *Tgfbr2* and *Smad3* promoters similar to, or higher than that of the known *Rev-erbα* target, *Bmal1* (Fig. 8C). In line with this finding, absence of *Rev-erbα* in brown preadipocytes leads to a ~15-fold induction of *Smad3* mRNA, and a tendency for higher *Tgfbr2* expression (Fig. 8D). These results demonstrate that *Rev-erbα* is a bona-fide transcription repressor of TGF-β pathway genes and loss of its repression leads to enhanced TGF-β signaling in brown adipocyte progenitors.

Collectively, findings from our study suggest a model in which *Rev-erbα* transcriptional control of the TGF-β pathway suppresses TGF-β activity, and attenuation of this inhibitory signal of brown fat development ultimately promotes brown adipocyte lineage determination and terminal differentiation (Fig. 8E).

## Discussion

Compare to the extensive knowledge of white adipocyte development, our current understanding of regulatory mechanisms governing BAT formation is only emerging. Employing genetic approaches and pharmacological interventions, our study uncovers a novel, cell-autonomous function of *Rev-erbα* as a key regulator of brown adipocytes lineage determination and terminal differentiation, which critically impacts brown adipose tissue development.

We found that *Rev-erbα* plays a critical role in promoting *de novo* brown adipocyte formation. In brown adipocytes, *Rev-erbα* exerts transcriptional repression of key genes of the TGF-β pathway, *Tgfbr2* and *Smad3.* TGF-β signaling is a known inhibitory pathway of brown fat development^30^,^31^,^32^. Various components of this pathway, including various ligands, the receptors or signal transducers, have been shown to suppress brown fat development. Ablation of Smad3, the ultimate signaling mediator of the TGF-β pathway, selectively induces brown-specific markers gene expression, particularly Prdm16, and enhances mitochondrial function^32^. Inhibition of the Activin receptor IIB or myostatin, both signaling through Smad3, also promote brown adipogenesis^30^,^31^,^36^,^37^. Interestingly, circadian clock regulators, such as *Bmal1* or the *Period* genes, have been demonstrated to modulate developmental signaling cascades, including the TGF-β pathway, in epidermal stem cells^33^. Thus, it is conceivable that the circadian clock regulatory circuit exerts coordinated temporal control on developmental signaling pathways to modulate stem cell activation and differentiation behaviors. Indeed, Smad3, the ultimate TGF-β signal transducer, is circadianly-regulated in mesenchymal stem cells^38^, while intrinsic diurnal phosphorylation of Smad3 are detected in suprachiasmatic nuclei^39^. Given the importance of TGF-β signaling in stem cell proliferation and differentiation^40^, *Rev-erbα* modulation of this pathway may confer specific temporal cues to fine-tune tissue development, such as in the brown fat. Our current study defines a specific role of *Rev-erbα* in brown adipocyte differentiation and development program. An intriguing aspect of its potential broader impact on additional TGF-β-regulated biological processes remains to be elucidated.

*Rev-erbα* has been shown to regulate white adipocyte differentiation^19^,^20^,^21^. Fontaine *et al.* reported that *Rev-erbα* promotes adipogenesis as a direct target of PPARγ^20^ and *Rev-erbα* agonist was found to stimulate 3T3-L1 differentiation to a similar extent as Rosiglitazone^25^, while Wang *et al.* found its dynamic regulation and bifunctional role in this process^21^. Hence, the precise mechanisms mediating *Rev-erbα* actions in white adipocyte differentiation remains unclear. Nonetheless, in line with the reported involvement of *Rev-erbα* in promoting white adipocyte differentiation^20^,^21^, we observed a severe loss of white adipose depot in *Rev-erbα*^*−/−*^ mice in addition to impaired BAT development (Fig. S1). Although *Rev-erbα* effect in other tissues could potentially contribute to this observed phenotype in WAT, our further analysis of stable silencing of *Rev-erbα* in 3T3-L1 found substantially suppressed adipogenesis (Fig. S2), indicating its cell-autonomous role in white adipocyte differentiation. TGF-β signaling is a potent inhibitor of adipogenesis^41^, and transgenic overexpression of TGF-β1 in mice led to a severe lipodystrophy-like phenotype with severe reductions of white and brown adipose tissue^42^. Therefore, it is intriguing to postulate that *Rev-erbα* modulation of TGF-β pathway may also contribute, at least in part, to its observed effect on white adipose tissue formation. The new evidence of *Rev-erbα* regulation of brown adipogenesis and its established effect on white adipose tissue imply that *Rev-erbα* participates in the adipogenic cascade, namely, the sequential activation events of adipogenic factors CEBPβ, PPARγ and CEBPα, a shared feature of brown and white adipocyte differentiation. As Smad3, the signal transducer of the TGF-β pathway, is known to directly repress the transactivation activity of C/EBP family of adipogenic factors to suppress adipogenesis^43^,^44^, *Rev-erbα* negative regulation of TGF-β pathway genes we observed is, therefore, in line with this notion.

Our study reveals a previously unappreciated function of *Rev-erbα* as a positive regulator of brown fat development. Not only the absence of *Rev-erbα* significantly impairs the *in vivo* formation and structural organization of brown fat, it results in substantial loss of brown fat characteristics. Nonetheless, due to the global targeted deletion nature of our current model, *Rev-erbα* functions in other tissues could potentially confound findings in BAT. Therefore, we focused on effects of *Rev-erbα* on BAT development in order to minimize such issues. Additional *in vitro* analysis and *ex vivo* studies of *Rev-erbα-*deficiency in brown adipocyte differentiation lends further support to cell-autonomous functions of *Rev-erbα* in brown adipogenesis. However, based on analyses of BAT in adult *Rev-erbα-null* mice, the severe impairment of embryonic and neonatal development could represent a developmental delay, as adult mice are able to generate functional brown fat (Fig. S3).

We detected a paradoxical induction of UCP-1 mRNA in adult Rev^*−/−*^ BAT, which was similarly reported by Gerhart-Hines *et al* previously^45^. However, due to the global ablation nature, we postulate that the significant lack of white adipose tissue observed in Rev^*−/−*^ mice may induce cold stress at ambient temperature (22 °C) leading to UCP-1 induction via sympathetic overdrive. We thus examined norepinephrine levels in plasma (Fig. S4A) and 16-hour overnight urine collection (Fig. S4B), and found doubling of the normal WT values in the Rev^*−/−*^ mice. This finding suggests activation of the sympathetic-adrenergic system and provides potential explanation why Ucp-1 is elevated in Rev^*−/−*^ BAT despite decreased brown adipogenic markers. It is also possible, that the comparable cold tolerance response observed between WT and Rev^*−/−*^ mice is a combined result of higher Ucp-1 expression together with reduced amount of brown fat in these mice. As Gerhart-Hines *et al*^45^ performed their analyses at thermo-neutral conditions, the sympathetic overstimulation observed in adult Rev^*−/−*^ mice in our study carried out under ambient facility temperature (22 °C) may account for certain differences in these independent studies. Given that the regulation of BAT formation and brown adipogenesis, which is the focus of our study, and acute modulation of its function, investigated by Gerhart-Hines *et al*^45^, both contributes to the total thermogenic capacity of BAT, these studies likely reflect distinct yet related aspects of *Rev-erbα* functions in BAT. Nonetheless, its specific role in brown fat activity will require future investigations using appropriate tissue-selective ablation models. Taken together, these newly discovered layers of *Rev-erbα* actions in BAT highlights its importance in fine-tuning thermogenic capacities during development and in response to functional demand.

As a major energy-dissipating organ, BAT activity critically regulates whole-body energy homeostasis^46^. As our study suggests, a *Rev-erbα*-controlled regulation of brown fat metabolic capacity could influence energy expenditure and metabolic homeostasis. Most importantly, as a ligand-activated nuclear receptor amenable to modulation by synthetic small molecule ligands^29^, *Rev-erbα* represents an ideal target for therapeutic interventions. Our elucidation of an important role of *Rev-erbα* in brown adipogenesis and the molecular mechanisms mediating its action may thus lead to discovery of new therapies against development of obesity and related metabolic disorders.

## Methods

### Animals

Mice were maintained in the Houston Methodist Hospital Research Institute mice facility at ambient temperature under a constant 12:12 light dark cycle, with light on at 7:00 AM (ZT0). All experimental protocols were approved by the IACUC animal care research committee of the Houston Methodist Research Institute, and carried out in accordance with the NIH Guidelines for the Care and Use of Laboratory Animals. Mice with global ablation of *Rev-erbα* were generated using gene targeted embryonic stem cells, Velocigene ES clone 11705A-E7, C57BL/6NTac origin, obtained from KOMP repository. Briefly, ES cell clones were injected into albino C57BL/6J-*Tyr* mouse blastocysts. The chimeric mice obtained were mated with C57BL/6J-*Tyr* mice for germ line transmission and the targeted *Rev-erbα* allele was confirmed by polymerase chain reaction (PCR) analysis using tail DNA. Offspring were backcrossed to C57BL/6J, and heterozygotes were bred to obtain homozygote null and littermate wild-type controls used in the study. The *Rev-erbα* homozygotes were born at a normal Mendelian ratio without obvious developmental defects. Tissue samples were obtained at ambient temperature at the same time of the day to ensure comparable circadian gene expression.

### Brown adipogenic differentiation of C3H10T1/2 and HIB1B cells

Mesenchymal stem cell line, C3H10T1/2, and brown preadipocyte cell line, HIB1B, were obtained from ATCC and maintained at subconfluence between passages in DMEM with 10% FBS and antibiotics. C3H10T1/2 differentiation to brown adipocytes was conducted as described^47^ without BMP7 pretreatment. Briefly, cells were grown to confluency and subjected to brown induction media of DMEM containing 10% FBS, insulin (20 nM), T3 (1 nM), isobutylmethylxanthine (0.5 mM), dexamethasone (5 mM), and Rosiglitazone (1 μM) for 3 days. Cells were then switched to maintenance media of DMEM containing 10% FBS supplemented with only insulin and T3 for 9 additional days. Fully differentiated brown phenotype with significant lipid accumulation and *Ucp-1* expression occur at 9 days (D9) after induction. Differentiation of HIB-1B and primary brown adipocytes were induced using similar cocktails with induction media for two days and maintenance media for four days.

### Generation of stable knockdown and overexpression cell lines

*Rev-erbα* shRNA construct in pGIPZ vector (V2LMM_25660) and scrambled control vector (RHS4346) were purchased from Open Biosystems, and mouse full-length *Rev-erbα* cDNA clone (MC203687 in pcDNA3 vector) was obtained from OriGene. C3H10T1/2 or HIB1B cell lines expressing *Rev-erbα* shRNA or cDNA were transiently transfected using FuGENE 6 reagent, and selection of stably transfected clones by Puromycin (for shRNA) or Neomycin (for pcDNA3) were started 48 hours following transfection and maintained for 7–10 days. The entire transfected plate was subcultured in order to minimize the effect of confluency on efficiency of differentiation, as described previously.

### Primary brown adipocyte, preadipocyte isolation and immortalization

Primary brown adipocytes and preadipocytes were isolated from interscapular brown adipose tissue pad of 4-week-old mice, as described^48^. Briefly, tissues were digested by Type I collagenase digestion in the presence of 1% BSA at 37 °C for 30 minutes. The suspension was filtered, centrifuged, and the top fat layer was collected as adipocytes and pellet containing the stromal vascular fraction was resuspended and plated. Preadipocytes in the stromal vascular fraction were passaged once prior to adipogenic differentiation. Immortalization of isolated primary preadipocytes was performed using by retroviral SV-40 Large T antigen transformation and puromycin selection, as described^47^.

### BODIPY and Mitotracker staining

BODIPY 493/503 (Life Technologies) staining of neutral lipids was carried out at a concentration of 1 mg/ml after formaldehyde fixation and incubation for 30 minutes. MitoTracker Red (MitoTracker Deep Red FM, 100 nM, Life Technologies) staining was applied to live cells at 37°C for 30 minutes before fixation to stain functional mitochondria, according to manufacturer’s protocol.

### RNA extraction and quantitative reverse-transcriptase PCR analysis

RNeasy miniprep kits (Qiagen) were used to isolate total RNA from snap-frozen tissues or cells. Tissues samples are collected at times as indicated and cell samples were obtained at non-synchronized normal culture conditions. cDNA was generated using q-Script cDNA Supermix kit (Quanta Biosciences) and quantitative PCR was performed using a Roche 480 Light Cycler with Perfecta SYBR Green Supermix (Quanta Biosciences), as described^49^. Relative expression levels were determined using the comparative Ct method to normalize target genes to 36B4 internal control or compared to controls as indicated.

### Immunoblot analysis

40–50 μg of total protein from tissues or cell homogenates were used for each sample on SDS-PAGE gel. After electrophoresis, protein were transferred to PVDF membrane, blotted using specific primary and secondary antibody and detected by chemiluminescence (Supersignal; Pierce Biotechnology), as previously described^49^. Smad3 phosphorylation in immortalized primary brown preadipocytes was assessed at 1 hour after indicated ligand treatment. The primary antibodies used were: Rev-erbα, PA5-29865 (Thermo Scientific), Bmal1, AB93806 (Abcam), UCP-1, AB3038 (Millipore); CEBPβ, sc-150, TBP, sc-204 (Santa Cruz); Smad3, 04-1035, phosphor-Smad3 (Ser 423/425), 07-1389 (Millipore).

### Chromatin immunoprecipitation (ChIP)-qPCR analysis

Immunoprecipitation was performed using Bmal1 Rev-erbα antibody (PA5-29865) or control rabbit IgG plus protein A/G beads, as described^50^. Briefly, cells were fixed by formaldehyde, lysed and sonicated to shear the chromatin. The immunoprecipitated chromatin fragments were eluted, treated with proteinase K and purified using Qiaquick PCR purification kit (Qiagen). Real-time PCR using Perfecta SYBR Green Supermix (Quanta Biosciences) was carried out with an equal volume (4 ul) of each reaction with specific primers. Sequences of the specific primers flanking the identified RORE sites are listed in supplementary Fig. S3. TBP were included as negative controls, and the known Rev-erbα target Bmal1 as a positive control. Data was expressed as fold enrichment over IgG after normalization to 1% input.

### Luciferase reporter assays

C3H10T1/2 cells were seeded to ~80% confluency overnight in 24 well plates. Transient transfection using FuGENE 6 (Roche) was carried out in four replicates, as described^39^. Per well, the transfection mixture contains 150 ng of TGF-β-responsive SBE4-Luc luciferase reporter (Addgene Plasmid 16495^51^) together with 20 ng of Renilla luciferase (pRL-TK, Promega) as an internal control. TGF-β1 (2 ng/ml) were added 16 hours after transfection and luciferase activity was measured using Dual-Glo luciferase assay system (Promega) 24 hours following ligand treatment. Reporter luciferase values were normalized to Renilla readings and expressed as fold of induction over controls.

### Statistical analysis

Data is expressed as Mean ± SE. Statistical differences between mean values of two groups were assessed by two-tailed, unpaired Student’s t test Student’s *t* test. P ≤ 0.05 is considered as statistically significant.

## Additional Information

**How to cite this article**: Nam, D. *et al.* Novel Function of Rev-erba in Promoting Brown Adipogenesis. *Sci. Rep.* **5**, 11239; doi: 10.1038/srep11239 (2015).

## Supplementary Material

Supplementary Information
