# Supplementary material for: Novel Function of Rev-erbα in Promoting Brown Adipogenesis
Source: Sci Rep. 2015 Jun 10;5:11239. doi: 10.1038/srep11239 (PMC4462032; doi:10.1038/srep11239)

Supplemental Information

Novel Function of Rev-erbα in Promoting Brown Adipogenesis

Deokhwa Nam[1], Somik Chatterjee[1], Hongshan Yin[2], Ruya Liu[3], Jeongkyung Lee[3], Vijay K. Yechoor[3], Ke Ma[1]*

[1]Center for Diabetes Research, Department of Medicine, Houston Methodist Research Institute, Houston, TX, 77030
[2]Department of Cardiovascular Medicine, Second Affiliated Hospital, Hebei Medical University, Shijiazhuang, 050017, Hebei, China.
[3]Diabetes and Endocrinology Research Center, Department of Medicine, Baylor College of Medicine, Houston, TX, 77030

Running Title: Rev-erbα regulation of brown adipogenesis

* To whom correspondence should be addressed: E-mail: kma@tmhs.org Phone: (713) 441 5084

Fax: (713) 793 7162

Figure S1.

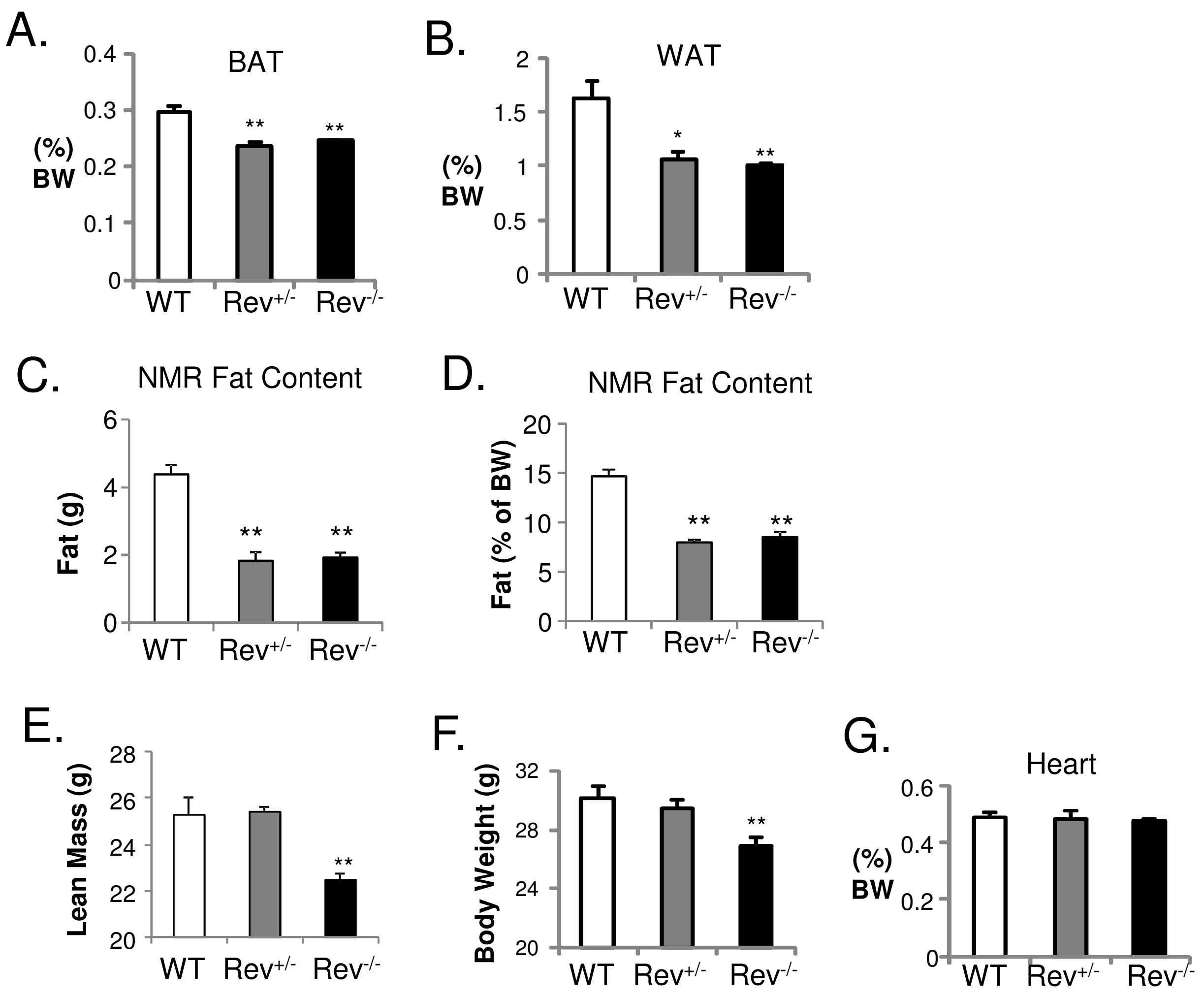

Figure S1. Adult *Rev-erbα*$^{-/-}$ mice exhibit reduction of white adipose tissue mass. (A) Reduction of interscapular brown adipose tissue mass in 10-week-old *Rev-erbα*$^{-/-}$ and *Rev-erbα*$^{+/-}$ as compared to WT mice (n=5-6/group). (B) Significant reduction of white adipose tissue in adult *Rev-erbα*$^{-/-}$ and *Rev-erbα*$^{+/-}$ mice as compared to WT mice, as analyzed by percentage of epididymal fat pad weight to body weight. (C-D) Reduction of total fat content as shown by NMR analysis of total amount (C), or percentage to body weight (D) in adult *Rev-erbα*$^{-/-}$ and *Rev-erbα*$^{+/-}$ mice (n=8-10/group). (E-F) Lower body weight in Rev-erbα-null mice (E), without altered heart weight (F). *, **: $P<0.01$ and 0.05 *Rev-erbα*$^{-/-}$ or *Rev-erbα*$^{+/-}$ vs. WT.

Figure S2.

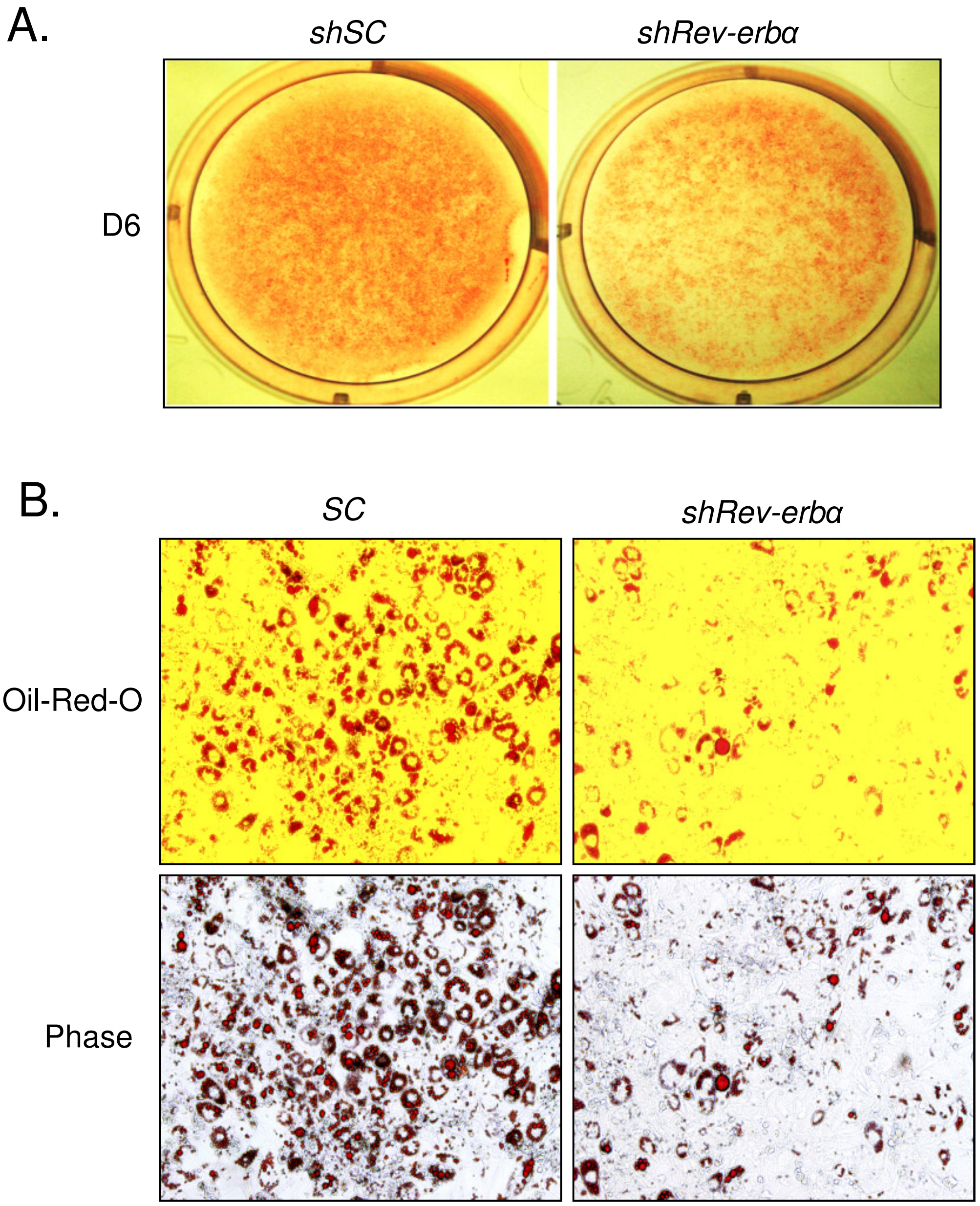

Figure S2. Stable silencing of *Rev-erbα* inhibits adipogenesis of 3T3-L1 preadipocytes. (A-B) Representative images of whole plate (A), or 10X magnification (B) of Oil-red-O staining of day 6-differentiated 3T3-L1 cells with stable expression of scrambled control shRNA (*shSC*) or *Rev-erbα* shRNA *(shRev-erba).*

Figure S3.

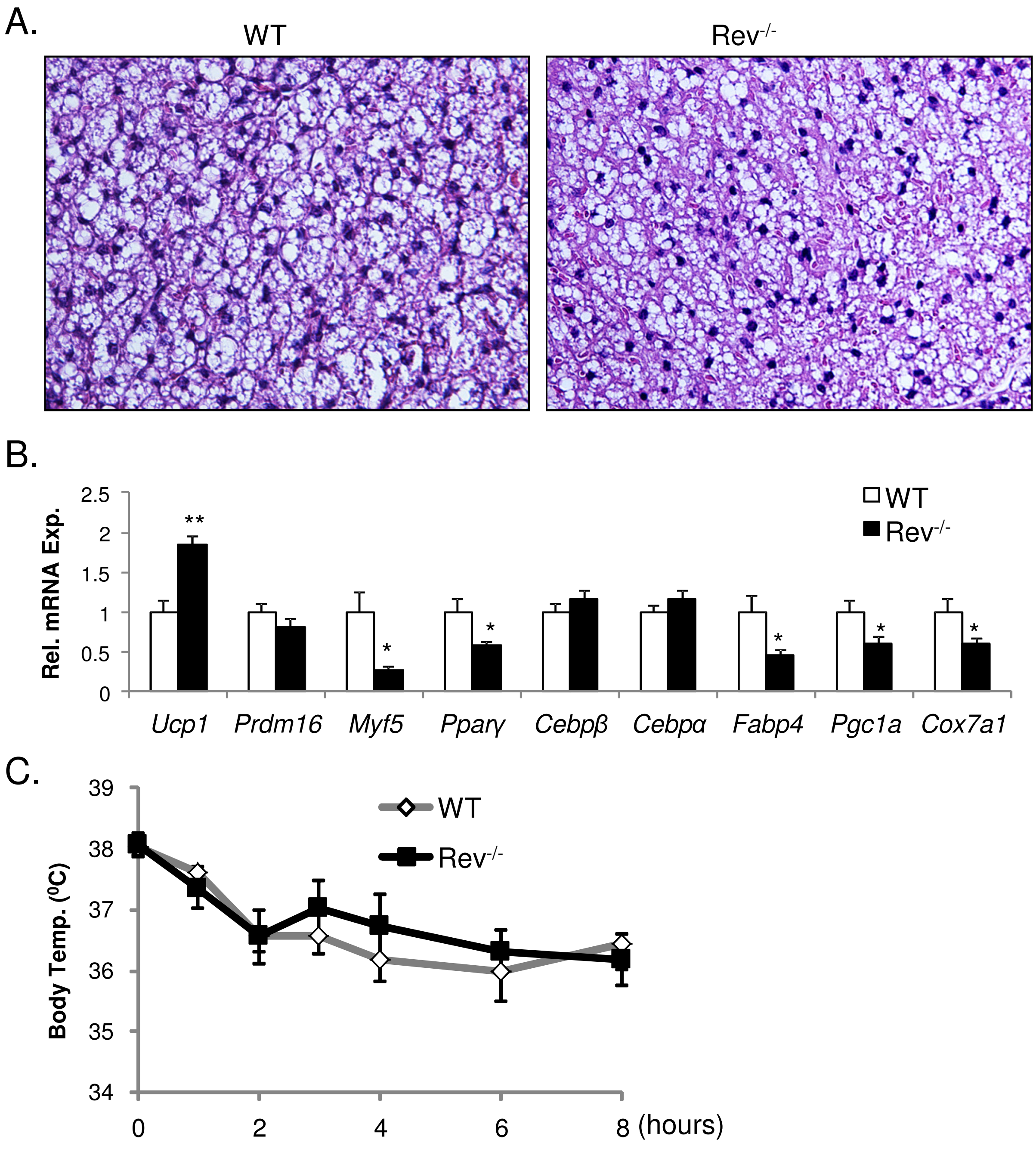

Figure S3. Analysis of brown adipose tissue in 10-week-old adult *Rev-erbα*$^{-/-}$ mice. Representative images of H/E histology of BAT (A), RT-qPCR analysis of brown fat gene expression (B), and cold tolerance test (C) of WT and Rev$^{-/-}$ mice (n=6/group). For cold tolerance test, mice initially maintained at ambient temperature (22^{0}C) were individually housed and subjected to 4^{0}C starting at 9AM and body temperature was monitored for 8 hours using a rectal probe. *, **: P<0.01 and 0.05 *Rev*$^{-/-}$ vs. WT.

Figure S4.

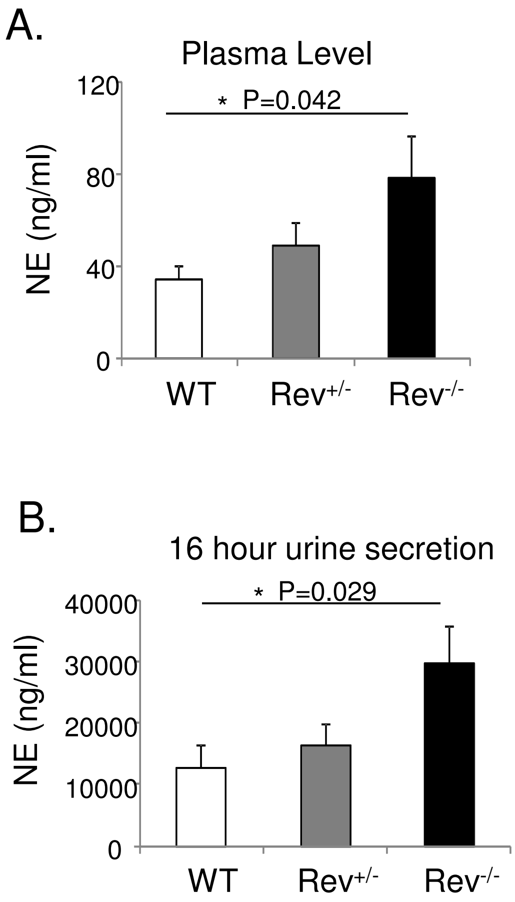

Fig. S4 Elevated plasma level and urine secretion of norepinephrine (NE) in $Rev^{-/-}$ mice. (A) Plasma levels of NE in 8-10 weeks old WT (n=9), Rev+/- (n=6) and Rev-/- (n=9) mice. Plasma was taken at 9am. (B) Total amount of NE level in urine of 8-10 weeks old WT (n=6), Rev+/- (n=4) and Rev-/- (n=6) mice collected for 16 hours from 5PM to 9AM overnight. The NE concentration in urine was analyzed and overnight secretion was calculated using the recorded total urine volume.

Figure S5. Rev-erbα binding sites identified on TGF-β pathway gene promoters.

Gene Name	TFBS Chromosome	TFBS Site Start	TFBS Site End	Gene Orientation	TFBS TSS Distance
Bmal1	Chr7	120351017	120351169	sense	38
Smad3	Chr9	63605957	63606109	antisense	156
TGFbr2	Chr9	116084431	116084583	antisense	-50

Figure S5. Analysis of *Rev-erbα* binding sites on proximal promoters of TGF-β pathway genes, including up to -2kb from transcription start site and the first intron. TFBS: transcription factor binding site; TSS: transcription start site.

Figure S6 . Primer sequences for ChIP-qPCR analysis of Rev-erbα binding sites on TGF-β pathway genes.

Genes		Sequences
Bmal1	Forward	CGATGCGGGTTTGACAGATA
	Reverse	GCACTCATTTCCGAACACAAC
Tbp	Forward	CCACACCCGCCACCAGTTCG
	Reverse	TACAGCCCGGGGAGCATCGT
Tgfbr2	Forward	GTCTGGGCTCCAAGTTCAAA
	Reverse	CGGGACTCTGAAGCTACATTTC
Smad3	Forward	GTTGCAGGAACTAGAGAGGATTAG
	Reverse	GTTCTGATCTCACCCAGCAG

Figure S7 . Primer sequences for RT-qPCR analysis.

Genes		Sequences
36B4	Forward	CGCTTTCTGGAGGGTGTCCGC
	Reverse	TGCCAGGACGCGCTTGTACC
Fabp4	Forward	AAGGTGAAGAGCATCATAACCCT
	Reverse	TCACGCCTTTCATAACACATTCC
Ucp1	Forward	TAACGGGTCCTCCCTGCCCG
	Reverse	CCGCGACTTCGGACTCCTGC
Pparγ	Forward	ACCGCCCAGGCTTGCTGAAC
	Reverse	TGGAGCACCTTGGCGAACAGC
Cebpα	Forward	GGTACGGCGGGAACGCAACA
	Reverse	CGGCTCAGCTGTTCCACCCG
Cebpβ	Forward	CAAGAGCCGCGACAAGGCCA
	Reverse	CTCGCGACAGCTGCTCCACC
Prdm16	Forward	ATGCACGCCGACTGCAGGAC
	Reverse	AAGGGCAGGCCTGGGGTGAA
Dio2	Forward	GATGGCTGGGCAGTGCCTGG
	Reverse	GGGCGGCAAGGAGAAACGCT
Smad3	Forward	CCCCCACTGGATGACTACAG
	Reverse	TCCATCTTCACTCAGGTAGCC
Tgfbr2	Forward	AGTGATGTCATGGCCAGCGAC
	Reverse	CGCAGACTTCATGCGGCTTCTC

Figure S8. Full-length images for immunoblot analysis of Fig.2C.

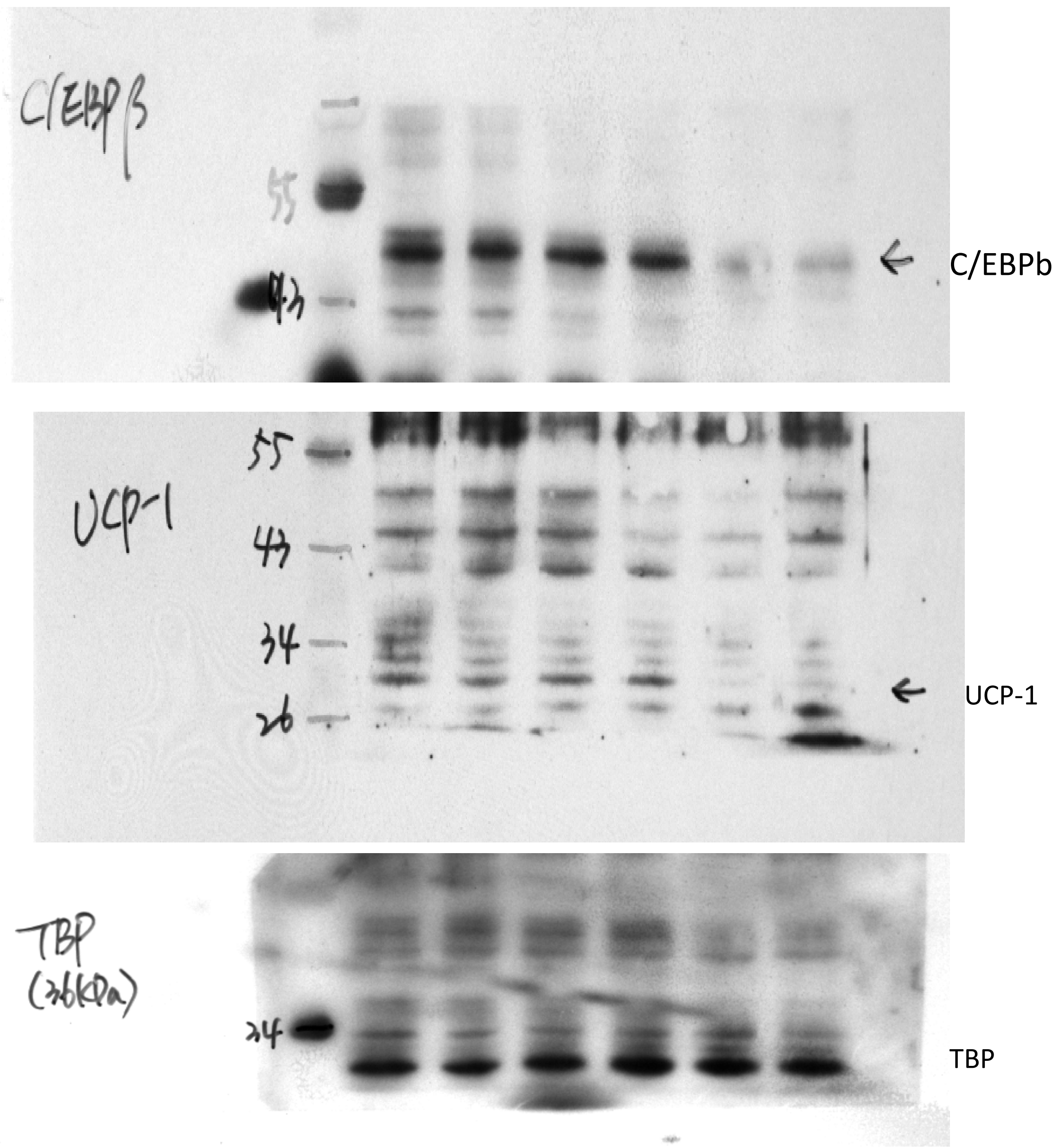

Figure S9. Full-length images of immunoblot analysis of Fig. 3A

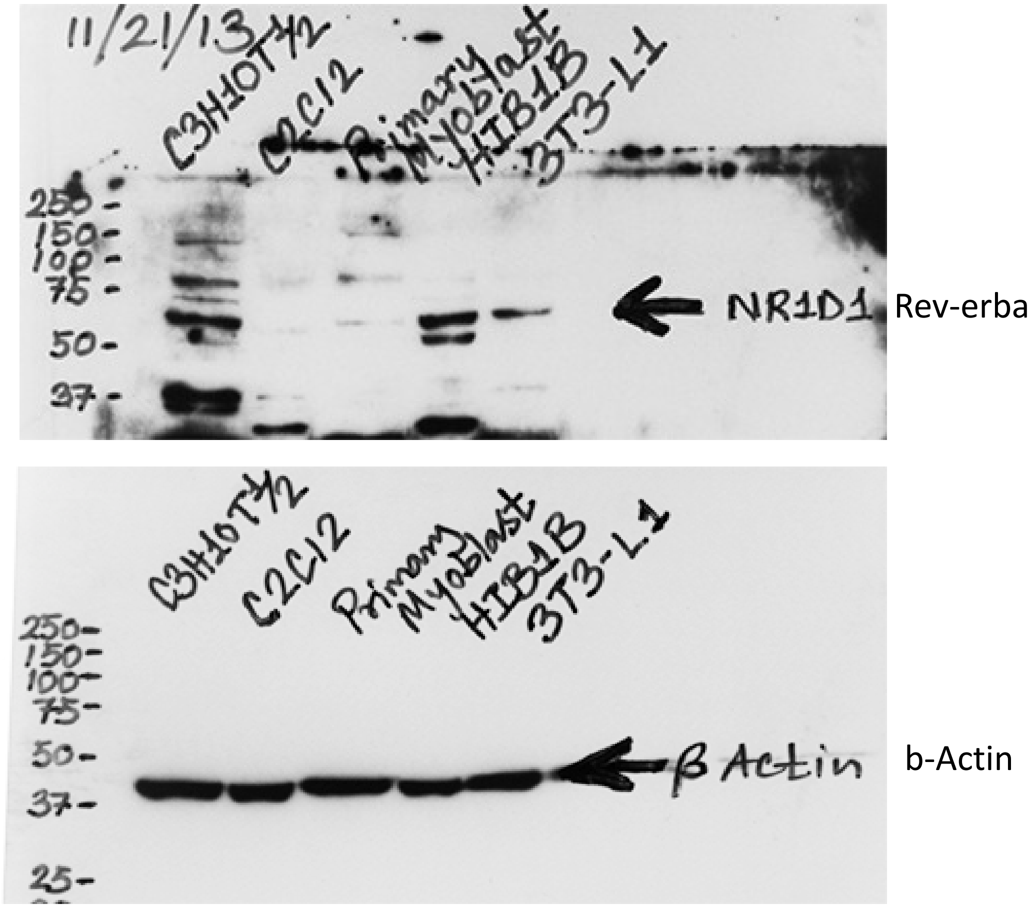

Figure S10. Full-length images for immunoblot analysis of Fig.8B.

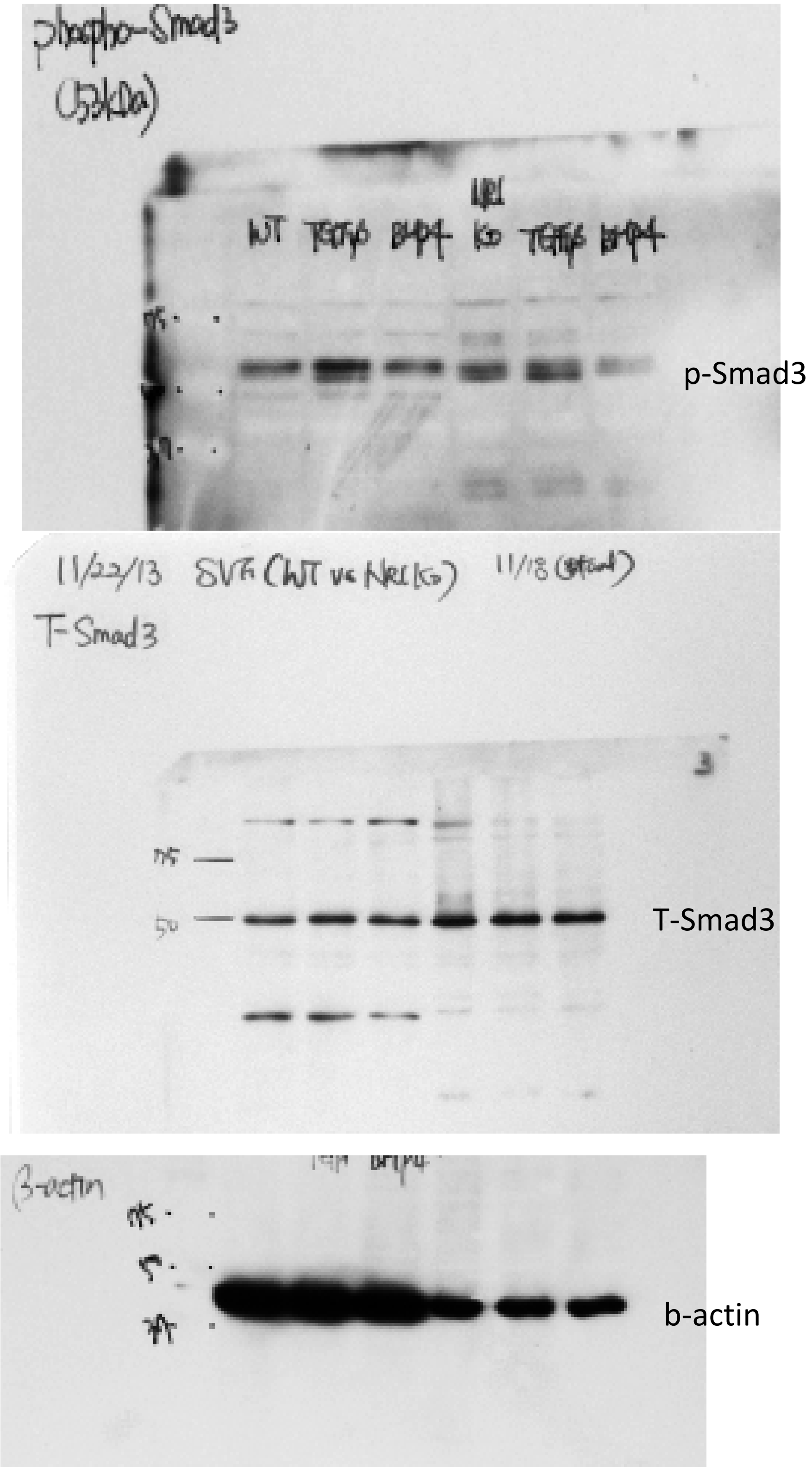

Supplement: Supplementary Information [file srep11239-s1.pdf]
